# Agro-Residues and Sucrose Alternatives in Confectionery Transformation Towards Glucose Spikes Minimization

**DOI:** 10.3390/foods14030491

**Published:** 2025-02-03

**Authors:** Snežana Zlatanović, Jovanka Laličić-Petronijević, Ferenc Pastor, Darko Micić, Margarita Dodevska, Milica Stevanović, Sven Karlović, Stanislava Gorjanović

**Affiliations:** 1 Institute of General and Physical Chemistry, Studentski trg 12/V, 11158 Belgrade, Serbia; snezana.zlatanovic@gmail.com (S.Z.); dmicic@iofh.bg.ac.rs (D.M.); 2 Faculty of Agriculture, University of Belgrade, Nemanjina 6, 11080 Belgrade, Serbia; jovankal@agrif.bg.ac.rs (J.L.-P.); mstevanovic@agrif.bg.ac.rs (M.S.); 3 Faculty of Chemistry, University of Belgrade, Studentski trg 12-16, 11000 Belgrade, Serbia; fpastor@chem.bg.ac.rs; 4 Institute of Public Health of Serbia «Dr Milan Jovanovic Batut», Dr Subotica Starijeg 5, 11000 Belgrade, Serbia; margarita_dodevska@batut.org.rs; 5 Faculty of Food Technology and Biotechnology, University of Zagreb, 10000 Zagreb, Croatia; sven.karlovic@pbf.unizg.hr

**Keywords:** apple, beetroot, carb:fiber, dietary fiber, low energy, pomace, jelly candies, antioxidant, anti-obesity

## Abstract

Apple and beetroot pomace flour (APF and BPF), along with two sweeteners, sucrose and a blend of sucrose substitutes (erythritol, stevia, inulin, and fructose), were simultaneously incorporated into three matrices formulated with agar, pectin, or gelatin as gelling agents. The aim was to produce jelly candies with high content of dietary fiber and dietary phenolics, and reduced energy value. The simultaneous incorporation of sucrose substitutes and pomace flour resulted in decrease of Carb:Fiber and Sugar:Fiber Ratio to extremely low values of 2.7–3.4 and 1.3–1.6 respectively, as well as in Energy:Fiber Ratio decrease to 9.2–12.3 kcal/g DF. Relative Antioxidant Capacity Index (RACI), as indicator of antioxidant potential, was calculated by assigning equal weight to Folin–Ciocâlteu, DPPH and FRAP assays applied upon in vitro digestion of 18 formulations of jelly candies. Results obtained for formulations with and without sucrose, as well as with and without APF or BPF, enabled insight into effects of pomace flour addition and sucrose substitution in each gelling matrix on functional properties. The incorporation and the substitution impact on postprandial glucose response were followed in vivo. Their superimposing resulted in glycemic index below 30 and low glycemic load. Efficiency of applied approach in functionalization of confectionery burden with energy and minimization of glucose spike represent an example of agro-residues re-introduction with the highest potential contribution to anti-obesity strategy.

## 1. Introduction

Agro-residues utilization in different food formulations enables avoiding economic loss and environmental issues [1]. Minimally processed agro-residues from fruit processing can be used directly as food ingredients, rich in dietary fiber (DP) and polyphenolics (DP) [2]. Restoration of DF and DP from agro-residues back to the food production can enhance the nutritional characteristics of processed food and accelerate the development of functional food products with targeted health-promoting properties, including low glycemic index (GI) products beneficial in protection of metabolic health and prevention of obesity and type 2 diabetes [2]. It is in accordance with FAO’s sustainable development goal 3: Ensure healthy lives and promote wellbeing for consumers at all ages [3].

Research focused on diversification of agro-residues re-introduction into confectionery production can facilitate development of up-cycled sweets with high fiber content. The adequate intake for fiber is 14 g total fiber per 1000 kcal [4]. Effect of regular intake of DF, critical for optimal metabolic health and the prevention of obesity, depends on the content of digestible carbohydrates, commonly very high in sweets. Optimal metrics to assess quality of diet or healthfulness of carbohydrate-rich products is based on carbohydrate-to-fiber-ratio (CFR) (grams of total carbohydrates consumed per gram of total fiber) [5]. A low CFR diet contributes to weight management and the preservation of metabolic health.

Large amounts of apple as well as beetroot pomace is generated annually in the juice industry (DOI: https://doi.org/10.3390/pr8030319), Rich in fiber and bioactive compounds, these underutilized by-products offer potential for sustainable use. Thus, they were chosen to demonstrate the feasibility of enriching confectionery with fruit and vegetable pomace. Prolonged supplementation with apple and beetroot pomace flour (APF and BPF), produced through a newly developed technology [6,7] has led to reductions in body weight and glycemia, along with improved glucose tolerance in mice on both high-fat, high-sucrose, and standard diets [8]. Cereal or dairy products functionalization by introduction of APF and BPF and their impact on the nutritional, technological and sensory characteristics related to increased content of fiber and polyphenolics were reported [9].

Jelly candy, a confectionery product burdened with energy and artificial additives, represents an appropriate model system to demonstrate possibilities for functionalization [10]. Jelly candies made with agar, pectin, and gelatin as gelling agents and sucrose as a sweetener were recently enriched with APF and BPF. Textural and sensorial properties, as well as polyphenolics and betalains retention were followed for nine months demonstrating good storability [11]. However, reported results showed carb to fiber ratio above 10 to 1 revealing the space for further enhancement of nutritional profile by partial or total replacement of sucrose.

Production of jelly candies with no sucrose added has been perceived as a challenge due to sucrose techno-functional properties (bulk sweetener, flavour enhancer and sustainer, food preservative). Having in mind popularity of jelly candies and raising level of obesity in all ages, including school-aged children and adolescents, this aspect seems important. Various alternative sweeteners were employed in the candies to replace free sugars intake [12,13,14] but until now successful incorporation of agro-residues in parallel with sucrose replacement was not reported.

The primary goal of this work was to optimize the composition of jelly candies by simultaneous introduction of the high amount of dietary phenolic (DP) and dietary fiber (DF) and low energy sweetener, in order to enable the minimization of postprandial glucose spike. The highest possible increase in DP and DF content was achieved by incorporation of agro-residues while the energy reduction by replacement of sucrose with appropriate combination of low-energy sweeteners. A randomized experimental setup was conducted utilizing three different gelling agents (agar and pectin for obtaining vegan jelly -candies and gelatin for comparative purposes) × 2 sweeteners (sucrose or combination of erithritol, stevia, fructose and inulin) × 3 levels of enrichment (two types of flour (APF and BPF) and control without any flour). Transformation of energy burdened confectionery into healthy products that meet the latest recommendations for adequate intake of DF as well as recommended CFR lower than 10:1 per energy value has been followed based on common in vitro analysis as well as in vivo surveying of postprandial glucose. The idea behind it-was to ensure health promoting properties of agro-residues to be available to all consumers, regardless of health status or life style, including those who avoid sucrose for any reason, even patients with impaired glucose control. The advantage of incorporation of whole, minimally processed pomace, as agro-residues rich in health promoting bioactive compounds, into low-energy matrix is discussed based on obtained results. The secondary goal of this study was to demonstrate applicability of indicators of anti-oxidant and anti-obesity potential in surveying of functionalization of food and estimation of its quality.

## 2. Materials and Methods

### 2.1. Apple and Beetroot Pomace Flour Preparation and Experimental Design

Apple and beetroot pomace, still classified in Serbia as a non-hazardous waste [15], were sourced from a local fruit processing plant (Fruvita doo, Smederevo). This pomace was the by-product of pressing whole, healthy, carefully selected, and washed apples and beetroots without any thermal or enzymatic treatment. The pomace was dehydrated on an industrial scale level, at a temperature below 55 °C, and grounded to the particle size of less than 300 µm. The final moisture content of the APF and BPF ranged from 4–8%, while water activity (Aw) was 0.2–0.4. Water activity was measured using the Novasina LabSwift Bench-model Water Activity Meter (Neutec Group Inc., 1 Lenox Avenue, Farmingdale NY, USA) at (25 ± 2) °C.

An experimental design was implemented, incorporating three gelling agents (agar, pectin and gelatin) and two types of pomace flour (APF and BPF) and 2 types of sweeteners (sucrose and combination of erythritol, stevia, inulin and fructose). Samples were taken from eighteen manufacturing batches (3 thickening agents × 2 sweeteners × 3 types of enrichment (with APF or BPF and without flour addition). Samples with sugar substitutes are marked with an asterisk.

### 2.2. Optimization of Jelly Candies Composition and Preparation

**Figure 1:**
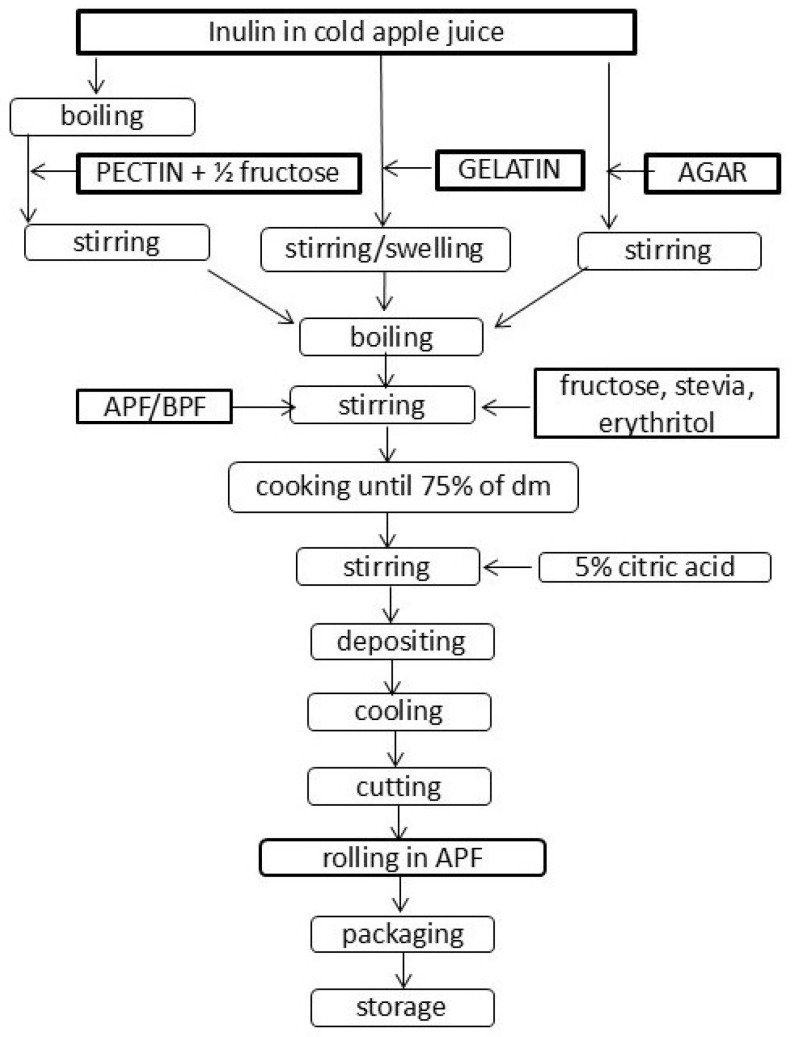
Jelly candies with sweeteners and APF/BPF addition processing scheme.

The amount of APF and BPF incorporated into jelly candies was optimized as well as composition of sweeteners. The preparation conditions were adjusted to preserve as many bioactive compounds present in APF and BPF as possible. Attention was paid to obtain products with desirable sensorial and textural properties. The pre-cooking procedures varied based on the gelling agent—agar powder from Gelidium algae (E406), beef gelatin, and pectin (E440)—as well as the type of sweetener used, either sucrose or an alternative sweetener blend. Patent application with claims protecting the process and the product was subjected to the Serbian intellectual property office [16]. Preparation procedure of jelly samples with sucrose and APF/BPF addition was described in detail in our previous research [13]. Since samples made with sucrose are presented in this research only for comparative reasons, here the scheme of jelly candies made with sweeteners and APF/BPF addition is given in Figure 1.

Controls were prepared in the same manner but without APF and BPF addition. Among others, the step that made the most significant difference in the preparation of jelly candies was the proposed new way of panning [11], which implies that the individual pieces obtained on the guitar wire cutter were rolled in APF instead of sugar, thus protecting the candies from external influences, while simultaneously additionally enriching them with DF.

### 2.3. Texture Analysis—Penetration Test

Analysis of mechanical properties was performed using texture analyser (Ta.HDPlus, Stable Micro Systems Ltd., Vienna Court, Lammas Road, Godalming, Surrey, UK) using 30 kg load cell. P/2 (steel, 2 mm diameter) probe was used for the full penetration in the samples. Probe speed was set at 2 mm/s. Based on the obtained data hardness was calculated as highest recorded force until break. Elasticity is measured from obtained force-distance curves as a distance from the starting point (contact of probe and sample) to the breaking point of the samples (in mm). The force-time curve generated during the test was also used to calculate the work of penetration. This was determined as the area under the obtained curve, using the software’s (Exponent 6.1, Stable Micro Systems Ltd., Vienna Court, Lammas Road, Godalming, Surrey, UK) numerical integration capabilities. All samples were tested in triplicate, and testing was conducted at 4 °C.

### 2.4. Determination of Proximate Composition and Calculation of Carbohydrates: Fiber, Sugar:Fiber and Energy:Fiber Ratio

The proximate composition was analyzed following the methods outlined by the Association of Official Analytical Chemists. Sucrose, D-glucose, and D-fructose were quantified using an enzymatic assay kit (R-BIOPHARM AG, Darmstadt, Germany). Total sugar content was calculated as the sum of glucose, fructose, and sucrose Total carbohydrates were determined using the formula: 100 − (fat (g) + ash (g) + moisture (g) + protein (g)).

Cellulose content was measured according to the standard method SRPS ISO 6541:1997 [17]. Fructan content was analyzed using the Fructan HK Assay Kit (Megazyme, Bray, Ireland). Soluble and insoluble dietary fiber (SDF and IDF) were determined using the enzymatic–gravimetric method (AOAC 991.43-1994(2000) [18]. Total dietary fiber was calculated as the sum of SDF, IDF, and fructan.

Energy value was calculated as the sum of carbohydrates multiplied by 4 kcal, lipids by 9 kcal, proteins by 4 kcal and DF by 2 kcal. CFR and SFR were calculated by dividing the total carbohydrates content and total sugar content by total DF content. EFR was calculated by dividing energy value expressed in kcal with DF content.

### 2.5. In Vitro Digestion and Determination of Antioxidant Activity of Digested Jelly Candies

In vitro digestion of jelly candies was conducted using the Infogest consensus method that simulates the oral, gastric and intestinal phases of human digestion [19] applied in triplicate on 18 produced jelly candies. A blank solution was prepared containing all reagents except the sample. The digested jelly candies were analyzed for total phenolic content (TPC) and antioxidant (AO) activity using spectrophotometric DPPH and FRAP assay.

#### 2.5.1. Determination of Total Phenolics

Total phenolic content was determined by Folin-Ciocalteu (FC) assay [20]. Measurements were done in triplicate. Results were expressed in gallic acid equivalent (GAE) per g of sample (µg GAE/g).

#### 2.5.2. Determination of AO Activity

The antiradical activity of samples against DPPH radical was measured by a modified method of Blois (1958) [21]. The results are expressed as µmol of Trolox equivalents per g of digested sample (µmol TE/g).

Determination of total reducing power by FRAP assay was performed according to the procedure previously described [11] with some modifications introduced- before the measurement the mixture was incubated at room temperature instead of 37 °C. The results were expressed as µmol Trolox equivalent per g of digested sample (µmol TE/g).

### 2.6. Calculation of Relative Antioxidant Capacity Index (RACI)

Central tendency was used to compare the AO activity of complex food samples determined by three assays (FC, FRAP and DPPH). Samples were ranked based on the mean value and standard deviation of the assays used. Due to differences in the units and the scale of the results obtained by application of various AO assays, the data in each dataset should be transformed into standard scores, dimensionless result of dividing the difference between the raw data and the mean value by the standard deviations. The standard scores of each sample for different assays are averaged giving a single unitless value entitled RACI. RACI is a specific combination of data from various AO methods with diffident units, without variance between them [22].

### 2.7. In Vivo Determination of Postprandial Glucose Response After Consumtion of the Same Amount of Pectine Based Jelly Candies

Ten non-diabetic adults (6 women and 4 men) aged 22–59 years, with a body mass index (BMI) of 19–22 kg/m^2^ and fasting glucose levels ≤ 6 mmol/L, were recruited for the study. All participants provided informed consent. The study was approved by the Ethics Committee of the Institute of Public Health of RS (Ethics approval number 5646/1, issued on 7 October 2022). Participants fasted for a minimum of 12 h and abstained from exercise, smoking, alcohol, and drug use. Postprandial glucose was determined after consumption of 25 g of jelly candies based on pectin, taken with 250 mL of water within 5 min. Six formulation based on pectin were tested and obtained results were compared. Blood samples were collected 5 min prior to jelly candy consumption (0 min) and at 15, 30, 45, 60, 90, and 120 min following consumption. Two tests were carried out per week. The glucose concentration was plotted over time, and the incremental area under the curve (IAUC) was calculated geometrically. This was done by summing the areas of the trapezoids over a 2-h period, excluding any area beneath the baseline fasting glucose concentration. The percentage decrease in IAUC was calculated in relation to the incorporation of APF and BPF, as well as sucrose substitution. Cross-comparisons were performed between samples containing APF and BPF, as well as against their respective control samples.

### 2.8. Determination of Glycemic Index and Load of Pectine Based Jelly Candies with APF and BPF Sweetened with Sucrose Alternatives

Determination of the GI of pectin based jelly candies enriched with APF and BPF and sweetened with sucrose alternatives was carried out in accordance with the ISO 26642:2010 standard [23]. Postprandial glucose was determined after consumption of 25 g of glucose or the amount of jellies containing 25 g of carbohydrates, consumed within 5 min with 250 mL of water. The GI was determined by calculating the average IAUC for each jelly, dividing it by the IAUC for glucose, and multiplying the result by 100. Glycemic load (GL) was calculated by multiplying the GI by the weight (in grams) of available carbohydrates in the jellies and dividing the product by 100 [23]. Significant differences were determined at *p* < 0.05.

### 2.9. Statistical Analysis

Statistical analysis of the data was conducted using XLSTAT (version 2014.5.03, Addinsoft, New York, NY, USA), analysis and statistics add-in for MS Excel (Microsoft Excel 2019 MSO, Microsoft Corporation, Redmond, WA, USA). The results were expressed as means ± standard deviation (SD). Significant differences between means were determined by one-, two- or three-way ANOVA with post-hoc Tukey’s HSD test (*p* < 0.05).

## 3. Results and Discussion

### 3.1. Compositional Optimization of Jelly Candies with Agro-Residues and Sucrose Substitutes

The development of a low-energy alternative to jelly candies was achieved by fortification with APF and BPF, replacement of sugar with low-energy natural alternatives and use of natural thickening and gelling agent. The introduction of fruit and vegetable pomace flour and the sucrose alternative were performed simultaneously to transform energy dense jelly candies into a nutritionally enhanced formulation. According to our knowledge, this is the first simultaneous application of whole pomace flour and alternative sweetening agents in the development of jelly products.

Compositionally optimized formulations with APF and BPF incorporated and sucrose replaced with mixture of natural low energy sweeteners were developed based on agar, pectin and gelatin as gelling agents. Direct employment of pomace allowed enrichment with anti-obesity factors while a specific manufacturing process enabled avoiding of their thermal deterioration and complete replacement of sucrose, both from the jelly matrix and surface. Ingredients ratio and jelly candies manufacturing process were adjusted to overcome challenging replacement of inner sucrose with natural sweeteners alternatives, associated with significant changes in texture, as well as replacement of superficial crystal sucrose with fine APF powder (<300 µm). The reports describe jelly candies sweetened with xylitole, erythritol, oligofructose syrup or combination of sucrose, stevia and xylitol [10] while erithritol, stevia, fructose and inulin were not combined in jelly candy until now.

Textural properties of developed jelly candies analyzed by penetration test were found acceptable. The use of gelatin contributes to a more pronounced elasticity, so, in a narrower sense, according to the nomenclature in the confectionery industry, it belongs to the hydrocolloids used to obtain gummy candies, while pectin and agar are usually used for jelly candies production, in addition to other gelling agents [24]. This is confirmed by our results (Table 1), because the samples with gelatin had more pronounced elasticity, compared to agar- and pectin—containing samples.

Although agar can give a firmer gel than pectin, in the present research, jelly candies with pectin showed slightly higher hardness values (Table 1). This is probably because the added pomace flours already contain pectin, which may cause the effective pectin concentration to be increased due to the high water binding capacity [25].

The two-way ANOVA results (Table S1) indicate that the addition of APF and BPF significantly impacts each of the measured textural parameters (Table 1). Work, hardness and elasticity showed highly significant *p*-values (*p* < 0.05) for all samples except pectin samples, suggesting that APF and BPF addition meaningfully influences the texture of the samples with gelatin and agar. The mean differences for work between the baseline sample A and variants containing APF or BPF were significant, while in case G the addition of BPF was not significant to the work parameter. This indicates that the APF and BPF additives greatly increase the work required, pointing to a stronger or more resistant texture. There are substantial increases in hardness across A samples with APF and BPF additions. However, in the sample with gelatin and BPF, hardness decreased. Elasticity showed fewer significant pairwise differences, which indicate that APF and BPF have less effect on this property compared to work and hardness.

### 3.2. Comparison of Proximate Composition of Developed Jelly Candies

Proximate composition of developed formulations was determined with the goal to evaluate effects of APF and BPF rich in fiber incorporation into six matrixes, based on agar, pectin and gelatin as gelling agents and two sweeteners, sucrose and mixture of its substitutes.

Due to their origin agar and pectin are in compliance with the growing demands of consumers for plant-based foods. Although gelatin is an animal-derived biopolymer it contributes to the protein content of the final products since it is a product of the structural and chemical degradation of collagen (Table 2C).

The nutritional parameters of APF are as follows: total carbohydrates: 86.18 ± 0.31 g/100 g, fats only 0.52 ± 0.10 g/100 g, and proteins 6.42 ± 0.05 g/100 g. Energy value are 317 kcal. APF are very rich in total fiber 29.22 ± 1.07 g/100 g (of which: insoluble fiber: 20.91 ± 0.59 g/100 g, soluble fiber: 8.22 ± 0.49 g/100 g, fructan: 0.10 ± 0.05 g/100 g); while the nutritional parameters of BPF are as follows: total carbohydrates: 75.22 ± 0.21 g/100 g, fats: 0.63 ± 0.10 g/100 g, and proteins 12.18 ± 0.26 g/100 g. Energy value are 303 kcal. BPF are also very rich in total fiber 26.08 ± 0.25 g/100 g (of which: insoluble fiber: 16.01 ± 0.31 g/100 g, soluble fiber: 6.62 ± 0.23 g/100 g, fructan: 3.45 ± 0.29 g/100 g).

The composition of jellies enriched with APF and BPF sweetened with sucrose substitutes, including moisture, protein, fat, carbohydrate, and fiber content, and counterparts sweetened with sucrose, as well as controls without APF and BPF were presented for each applied gelling agents separately, in Table 2. Content of DF in all jelly candies with pomace and sucrose substitutes developed within the scope of this study exceeded by far values for gelled products reported in literature [26] as well as requirement to claim high fiber (at least 6 g of fibre per 100 g or at least 3 g of fibre per 100 kcal) [27]. In jellies with APF and BPF sweetened with sucrose DF content was higher than 3 g per 100 g of product (3.7–4.9 g per 100 g) enabling claim “fiber source” to be reached [11]. Due to presence of inulin in sweeteners mixture which is superimposed with DF originating from APF and BPF, DF value was much higher than 6 g per 100 g of product as necessary to declare as high fiber foods. According to our knowledge, DF content of 22.2 to 27.6 g per 100 g is the highest reported in jelly candies until now. The fiber content in the control samples depended on the gelling agent used (*p* < 0.0001, Table S2). It was low in the case of gelatin (0.17 ± 0.1) and agar (0.6 ± 0.2) while in the case of pectin it reached 2.2 g per 100 g of products.

Jelly candies based on agar with APF and BPF sweetened with erythritol, stevia, inulin and fructose (A*-APF and A*-BPF) had a much lower sucrose content (3.0–6.3 g/100 g) than respective control without pomace addition (49.3 g/100 g), and jelly candies with APF and BPF sweetened with sucrose (45.9–49.4 g/100 g). A*-APF and A*-BPF contain higher amounts of fructose (30.1–26.8 g/100 g) compared to control and jelly candies with APF and BPF sweetened with sucrose. BPF contains fructan, which is more reflected in the difference in the composition of jelly candies sweetened with sucrose, than with alternative sweeteners including inulin. The amount of inulin added is so high that the contribution of inulin inherently present in BPF is almost negligible. In contrast, the higher sucrose content in BPF compared to APF is reflected only in samples without sucrose. Isocaloric replacement of other carbohydrates with fructose results in clinically significant improvements in glycemic control, without affecting insulin in people with diabetes [28,29]. Although the protein content of jelly candies with APF and BPF is several times higher than the control without pomace, its amount is not significant and represents 2 to 4% of the energy value from protein.

### 3.3. Carb:Fiber, Sugar:Fiber and Energy:Fiber Ratio as Indicators of Anti-Obesity Potential

The carbohydrate quality metrics was recently proposed [5,30]. It is defined as ratios of carbohydrates-to-fiber. For each 10 g of total carbohydrates in a diet or product, there should be at least 1 g of fiber (10:1). The fiber scoring system was based on the American Heart Association recommendations to consume 1 g of fiber for every 10 g of carbohydrates while the other three variants introduce a free sugar threshold based on the WHO recommendations. The American Heart Association issued a recommendation to identify healthy cereal-based products based on the total carb—fibre ratio, but such metrics has not been applied on confectionery yet.

A simple metrics utilizing carbohydrate to fiber ratio (CFR), recommended to be 1 to 10 for processed food, as well as sugar:fiber ratio (SFR) [5] was applied to evaluate APF and BPF as ingredients whose addition can contribute significantly DF content without increasing sugar content [11]. Low CFR (below 2) ascribed to both APF and BPF as well as low SFR indicated high potential of agro-residues to enrich food with DF.

**Figure 2:**
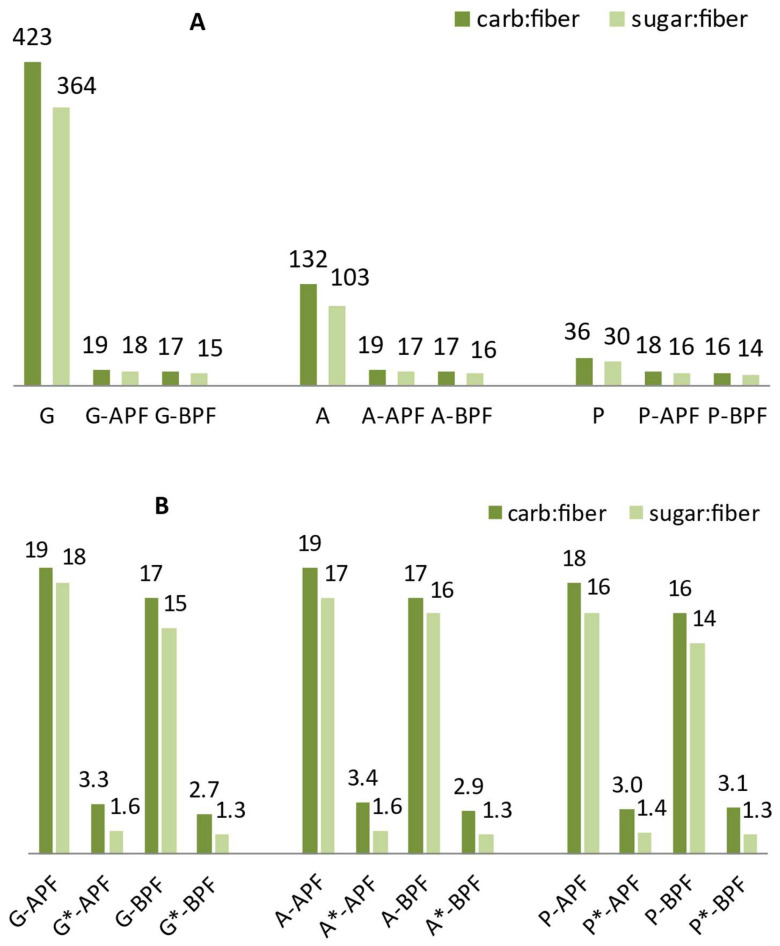
Effect of APF and BPF introduction in matrix with sucrose (**A**) and its subsequent replacement with low energy sweeteners (**B**) on carb:fiber and sugar:fiber ratio. Samples with sucrose replaced are marked with *.

The same metrics was used to estimate enhancement of jelly candies nutritional profile by pomace incorporation and sucrose substitution. Values of CFR and SFR of nine formulations without sucrose are presented on Figure 2A. Being almost depleted of DF control samples with sucrose based on gelatin and agar had very high values of CFR (423 and 132) and SFR (364 and 103), respectively. Pectin based jelly candies had CFR and SFR 36 and 30 respectively. The incorporation of APF and BPF into gelatin and agar matrices sweetened with sucrose reduced CFR and SFR by approximately 95 and 85%, respectively, while in pectin, these ratios were reduced by approximately 50%. The ratio between 19 and 14 ascribed to jelly candies with APF and BPF sweetened with sucrose indicated improvement in functionality related to DF enrichment. However, ratio of 10 to 1 was not reached. The simultaneous incorporation of sucrose substitutes and pomace flour enabled additional remarkable increase in DF and a decrease of CFR and SFR to extremely low values of 3.4–2.7 and 1.3–1.6, respectively (Figure 2B). CFR and SFR were reduced by approximately 80 to 90% compared to counterparts with sucrose.

Ratio of control samples with alternative sweeteners, depleted of various bioactive compounds presented in APF and BPF, was below 10:1. Reduction of CFR and SFR with addition of APF or BPF, in comparison to control samples, was approximately from 10 to 30%. CFR for gelatin, agar and pectin based controls was 4.2, 3.9 and 3.6 while SFR was 1.9, 1.8 and 1.7, respectively. A high amount of inulin enabled more than satisfactory fiber content, however, the addition of APF or BPF introduced bioactive polyphenolics, which can be detected using standard spectrometric methods for AO activity determination.

It is evident that the carbohydrate-to-fiber ratio (CFR) and sugar-to-fiber ratio (SFR) can be used to track the enhancement of the nutritional profile associated with the use of underutilized agro-residues or other plant based matrices rich in dietary fiber.

Energy to fiber ratio (EFR) can be applied to differentiate products according to energy input. The ratio reflects energy input per each gram of fiber (Table 3). As can be seen, lower EFR is ascribed to jelly candies with sucrose replacement than to corresponding counterparts or controls with sucrose.

Jelly candies with such high fiber content can significantly contribute recommended intake of 14 g DF per each 1000 kilocalories (4184 kJ) [4] i.e., less than 71.4 kcal per gram of DF. The range of 80.5 to 61.6 attributed to jelly candies with APF or BPF aligns well with this recommendation (Table 3). The extremely low ratios ascribed to jelly candies with APF or BPF along with sucrose substitutes (12.3 to 9.2 for BPF), serve as very strong indication of the product applicability in an optimal diet and its potential to prevent metabolic disorders associated with a high energy diet or low fiber intake. The fiber content in jelly candies with beet pomace is higher than in those with apple pomace. As a result, the energy value is lower in jelly candies containing beet pomace, which accounts for the difference in EFR.

Attention should be given to the development of such products, especially those based on underutilized agro-residues, due to their potential to simultaneously improve public health and support a circular approach in the fruit and vegetable processing industry. Also, the importance of applying simple indicators such as CFR, SFR and EFR to support consumers who face a huge variety of products labeled as whole grain, integral or fiber rich but actually burdened with energy, needs to be highlighted. As simple and informative indicators, CFR, SFR and EFR should be considered as candidates to be declared alongside the composition of product.

### 3.4. Total Phenolic Content and AO Activity

There is an increasing number of scientific evidence that a plant-based diet can be effective not only in preventing type 2 diabetes but also in improving its management [31]. At the same time, individual nutrients are not responsible for this, but rather a diet that includes an increased intake of fiber, antioxidants and anti-inflammatory nutrients such as polyphenols [32].

**Figure 3:**
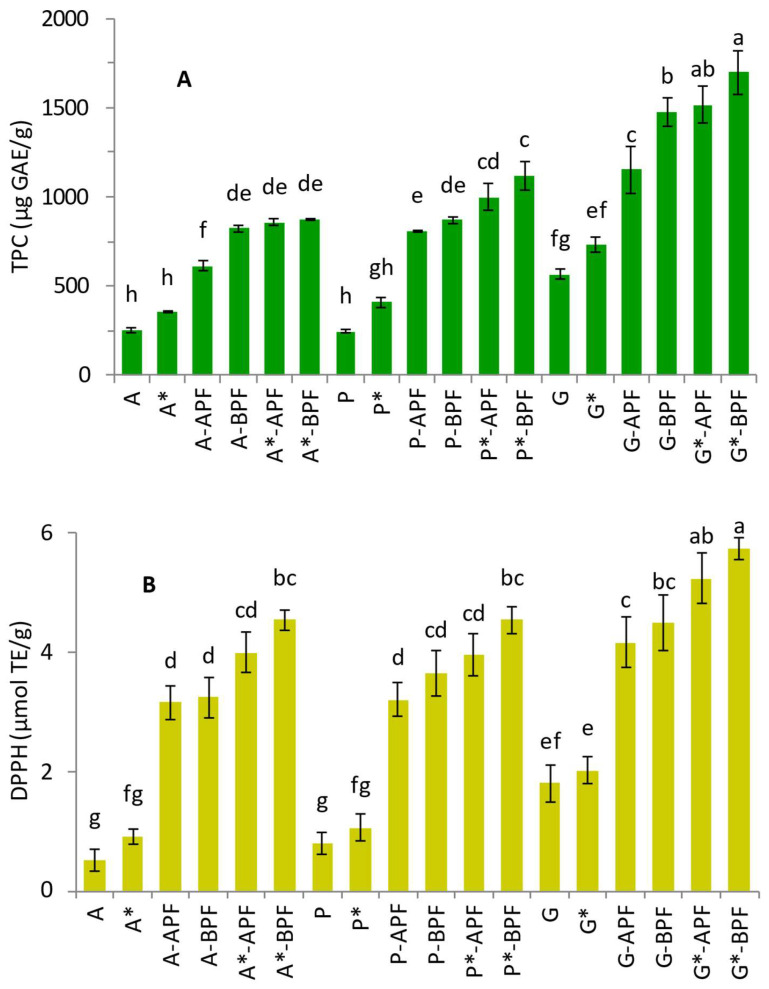

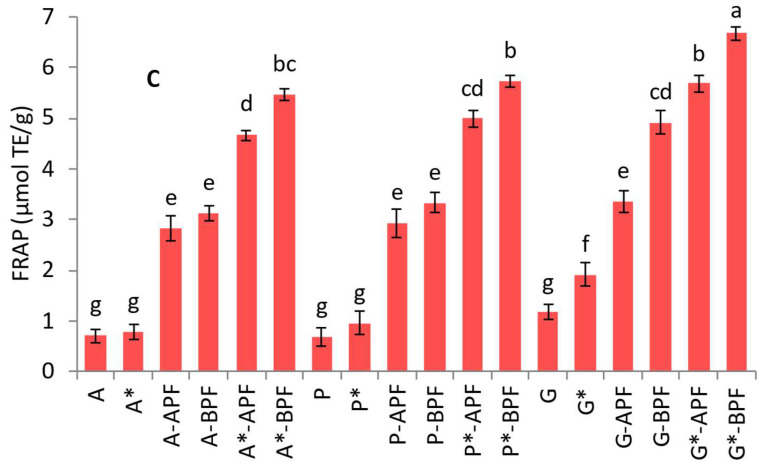
(**A**) Total phenolics (TPC) and AO activity determined by (**B**) DPPH and (**C**) FRAP assay of jelly candies sweetened with sucrose or mixture of sucrose alternatives (*) digested according to INFOGEST procedure. Data were subjected to three-way ANOVA (Table S3, factors: Flour—tree levels: Control, APF and BPF degree of freedom was 2; Sweetener—two levels: NO-sugar* and sugar degree of freedom was 1; and Thickening agent—three levels: A, G and P degree of freedom was 2; interactions—“Flour × Sweetener” degree of freedom was 2; “Flour × Thickening agent” degree of freedom was 4; “Sweetener × Thickening agent” degree of freedom was 2; “Flour × Sweetener × Thickening agent” degree of freedom was 4). Different lowercase letters indicate a significant difference of means, according to Tukey’s HSD test (*p* < 0.05).

In contrast to fiber such as inulin, fruit or vegetables pomace has potential to enrich food with DF and a more significant amount of DP having anti-inflammatory, anti-obesity, anti-diabetic and AO activity [33,34,35]. Increase in total DP related with pomace introduction into jelly candies was followed using Folin-Ciocialteu assay while the AO activity was determined using FRAP and DPPH assays. All assays were applied after in vitro digestion of jelly candies. High content of DP along with prominent AO activity was noticed in jellies enriched with APF and BPF (Figure 3). Control samples with apple juice added but without APF or BPF exhibited moderate levels of TPC and AO activity. The inclusion of APF and BPF, however, led to a significant increase in both TPC and AO activity (Figure 3A). Phenolic content was higher in samples with sucrose substitute than with sucrose due to contribution of natural sweeteners (*p* < 0.0001, Table S3).

A similar pattern was observed across all applied assays (Figure 3). The lowest values were recorded in the control samples, followed by those with APF, while the highest values were noted in samples containing BPF. Notably, lower values were consistently observed in sucrose-sweetened samples compared to their counterparts prepared with sucrose substitutes. However, this difference was not statistically significant in the case of the control samples. The highest values were found in BPF samples sweetened with sucrose substitutes, particularly in gelatin-based (Figure 3). While the matrix effect of agar and pectin were found similar, gelatin contributed more significantly to TPC and AO activity (*p* < 0.0001, Table S3). The gelatin itself is known to exhibit some level of AO activity.

### 3.5. Relative Antioxidant Capacity Index as Indicator of Anti-Oxidative Potential

**Figure 4:**
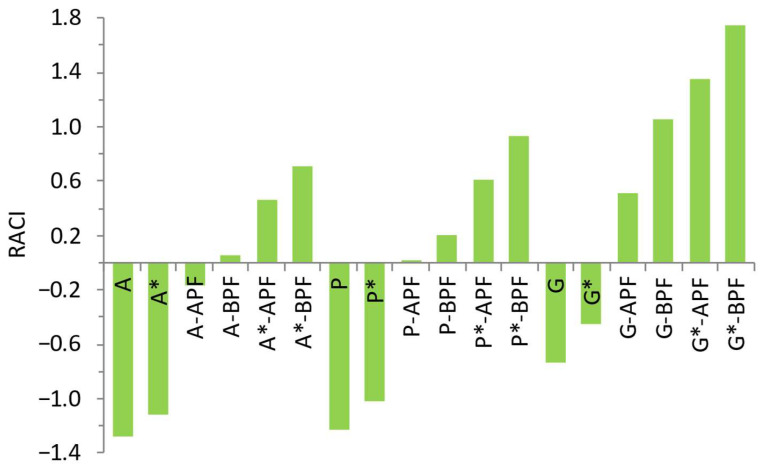
Ranking of RACI values in agar (A), pectin (P) and gelatin (G) jelly candies enriched with APF and BPF, sweetened with sucrose and sucrose alternative mixture (marked with *), in comparison with respective controls.

Here, the Relative Antioxidant Capacity Index (RACI) was applied as an indicator of jelly candies functionalization in terms of increased AO potential. RACI is calculated by assigning equal weight to FC, DPPH and FRAP [22]. Since it captures total reducing ability, the FC assay is considered an additional AO assay. RACI enabled a comprehensive comparison between the nine samples developed within the scope of this study and their nine sucrose counterparts. As a relative index, RACI provided an accurate ranking of the AO capacity for all jelly candies considered. The ranking of 18 formulations, which included six controls (three with sucrose and three without) and formulations enriched with APF and BPF (six with sucrose and six without), provided insight into the effects of flour addition and sucrose substitution. The RACI ranking is presented in Figure 4. As seen, the lowest, negative values of RACI were ascribed to all controls with sucrose. The highest values of RACI were ascribed to jelly candies with BPF based on gelatin. When comparing APF and BPF counterparts based on the same gelling agent and the same sweetener, those with BPF showed superior RACI values compared to those with APF. Also, formulations with sucrose substituted had higher RACI values than those with sucrose, with the highest value obtained in these samples. Contribution of inulin and stevia are probably responsible for higher values of RACI of jelly candies with sucrose substitutes. The matrix influence was found to be fairly consistent. Samples based on gelatin as gelling agent have the higher values of all parameters measured. As a result, their RACI values were significantly higher compared to those with agar and pectin (Figure 4).

Both measured and calculated parameters were correlated using regression analysis. Stepwise regression between RACI and the used AO assays (FC, FRAP and DPPH) revealed that each of the three assays matched RACI, and that correlations between RACI and the used assays were highly significant (*p* < 0.01 level).

### 3.6. Postprandial Glucose Response of Pectine Based Formulations

The low CFR and SRF of the developed formulations demonstrated the potential of pomace flour incorporation to support glucose control, which was further examined by comparing postprandial glucose responses to assess the effects of simultaneous or individual sucrose replacement and pomace flour addition. Pectin based candies were chosen for further analyses due to the lowest values of SFR and CFR as well as plant origin of gelling agent. Postprandial glucose responses to six samples of pectin based jelly candies were tested during three weeks. Jelly candies with APF and BPF, with and without sucrose, were compared to controls with and without sucrose. In the preliminary in vivo experiment, the participants consumed an equal amount of six different pectin formulations. For each of the six samples, the incremental area under the curve (IAUC) for capillary blood glucose concentration over 120 min was calculated. Based on obtained values both the addition of APF and BPF and sucrose replacement was associated with significantly improved glucose tolerance. The difference in IAUC values for P and P* (137 and 55 mM × min) showed that sucrose substitution resulted in 60% IAUC reduction. The reduction related to sucrose substitution was found similar to the reduction caused by flour incorporation. The incorporation of APF and BPF resulted in IAUC decrease to 61 and 55 mM×min (P-APF and P-BPF) and 24 and 22 mM × min (P*-APF and P*-BPF). In comparison to P and P* the reduction was 55–60%. Samples P*-APF and P*-BPF had 82 and 84% lower IAUC than P. The consistent difference between BPF and APF observed could be related to lower CFR, SFR and EFR of BPF, as well as higher TPC but it was not statistically significant.

### 3.7. Glycemic Index and Load of the Jelly Candies Based on Pectin

**Figure 5:**
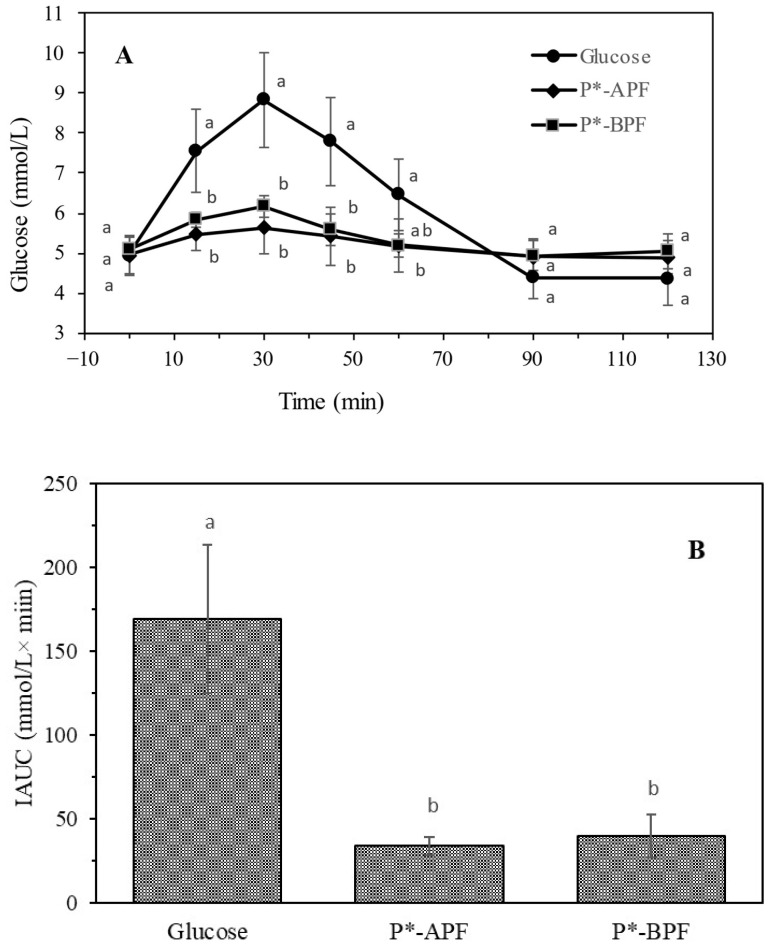
OGTT curve (**A**) and OGTT IAUC (**B**) after consumption of 25 g of glucose or jelly candies enriched with APF and BPF (P*-APF and P*-BPF) and sweetened with sucrose substitutes containing the same content (25 g) of available carbohydrates. OGTT data were subjected to two-way repeated-measures ANOVA (Table S4; between-subjects factor: Flour; three levels: Glucose, APF, BPF, degree of freedom was 2; within-subject factor: Time; seven levels: 0, 15, 30, 45, 60, 90 and 120 min; degree of freedom was 6; interaction “Flour × Time”; degree of freedom was 12). IAUC data were subjected to one-way ANOVA (Table S5; factor: flour—tree levels: Glucose, APF and BPF degree of freedom was 2). Different lowercase letters within the same time (**A**) indicate a significant difference of means, according to Tukey’s HSD test (*p* < 0.05).

The GI of the jelly candies based on pectin with the lowest carb:fiber ratio was determined. Blood glucose levels rose noticeably 15 min after consuming glucose, whereas the rise was significantly lower and more stable following the consumption of jelly candies with APF and BPF, sweetened with a sucrose substitute (Figure 5). The peak blood glucose level upon consumption of glucose was highest after 30 min. In contrast, both jellies containing APF and BPF caused a significantly smaller increase during the first hour and returned to baseline before glucose. By 120 min, blood glucose levels in all cases had returned to baseline.

The curve and area under the curve (AUC) of the oral glucose tolerance test (OGTT) demonstrated a significantly improved glucose tolerance of both jelly candies in comparison to the glucose group. Based on the calculated GI, both jellies were classified as low-GI foods (below 30). Additionally, they were categorized as low GL foods. This data indicates that the developed jellies have a significantly higher intake allowance compared to commercial versions. Values for commercially available jellies GI varied but they are commonly very high. The International Table of Glycemic Index confirms that jelly candies belong to foods with a high GI [32,36]. The GI of jelly candies, where sucrose was partially or fully replaced with maltitol and erythritol, was assessed through a prospective crossover study, was 81.9, 54.1 and 49.9 respectively [37]. Pectin based jelly candies enriched with APF and BPF and sweetened with sucrose belong to the low GI category [11], but with almost twice higher IAUC and GI than counterparts sweetened with sucrose alternatives. In conclusion, jelly candies obtained within the scope of this study are a good carriers for compounds with anti-obesity effect and GI lowering potential. They present a promising option for reducing blood glucose spikes [38].

Fiber causes negligible direct blood glucose response [36]. The higher percentage of DF corresponds with slower digestion and a lower increase in glucose since fiber participates in the mechanism responsible for the glucose retardation effect that may markedly reduce the access of glucose to the epithelium. Presence of inulin is considered important in terms in GI decrease [39]. Recent analyses confirmed that main glycemic indicators such as fasting blood glucose, glycosylated hemoglobin HbA1c, fasting insulin and homeostasis model assessment-insulin resistance were significantly reduced by fructan supplementation, particularly in the prediabetes and type 2 diabetes populations. Evidence supports that administration of fructan supplementation may have potential clinical value as an adjuvant therapy for prediabetes and type 2 diabetes management [39].

Jellies enriched with pomace flour and sweetened with sucrose substitute satisfy the criteria to fit into dietary guidelines for diabetic and obesity prevention (high fiber intake and low GI) [40]. Although the general perception is that jelly candies raise blood sugar levels, the results obtained confirmed that those with the right formula can control glycemic response and minimize blood glucose spikes.

## 4. Conclusions

The simultaneous incorporation of agro-residues and the replacement of sucrose with low-energy substitutes have led to the development of innovative jelly candy formulations with enhanced nutritional profiles. This enhancement was effectively measured using carbohydrate-to-fiber, sugar-to-fiber, and energy-to-fiber ratios, alongside the Relative Antioxidant Capacity Index (RACI), and was confirmed through in vivo assessments of postprandial glucose responses. The resulting formulations, which incorporate fruit and vegetable pomace, significantly increased dietary phenolics (DP) and dietary fiber (DF) intake without contributing excessive calories. By introducing minimally processed pomace into entirely natural matrices, free from artificial additives and sucrose, the potential to transform traditional jelly candies into healthier alternatives that align with current dietary recommendations was demonstrated. These innovative, sugar- and gluten-free jelly candies, rich in DF and DP, with extremely low GI and GL, represent a viable alternative to conventional sugar-based confectioneries. The difference between inclusion of BPF and APF in terms of GI -was not shown to be significant. Both flour—evinced high DF content and various DP with GI lowering power, whose effects are superimposed. This functional approach can effectively mitigate glucose spikes and contribute to public health initiatives aimed at obesity prevention while supporting environmental sustainability through the utilization of agro-residues. Future research will focus on diversifying these products further by incorporating additional natural sweeteners and conducting comprehensive sensory analyses with a larger consumer base to optimize formulations.

## Figures and Tables

**Table 1:** Textural properties of jelly candies based on agar (A*-APF and A*-BPF), gelatin (G*-APF and G*-BPF) and pectin (P*-APF and P*-BPF), with APF and BPF sweetened with erithritol, stevia, inulin and fructose in comparison with respective controls without pomace addition (A, G and P) obtained by penetration test.

	A*	A*-APF	A*-BPF	G*	G*-APF	G*-BPF	P*	P*-APF	P*-BPF
Work (mJ)	312.3 ± 62.6 ^e^	1653.2 ± 219.7 ^c^	1044.1 ± 151.7 ^d^	1997.2 ± 94.5 ^b^	2936.0 ± 87.0 ^a^	2031.1 ± 43.5 ^b^	1577.7 ± 27.0 ^c^	1473.3 ± 119.6 ^c^	1396.8 ± 137.5 ^c^
Hardness (g)	23.9 ± 4.7 ^e^	117.7 ± 17.0 ^bc^	76.2 ± 11.0 ^d^	246.6 ± 23.4 ^a^	235.5 ± 0.2 ^a^	145.7 ± 2.2 ^b^	112.6 ± 0.4 ^c^	106.9 ± 6.3 ^cd^	97.7 ± 11.3 ^cd^
Elasticity (%)	9.1 ± 0.8 ^ab^	9.5 ± 0.6 ^ab^	9.9 ± 0.1 ^a^	10.0 ± 0.0 ^a^	10.0 ± 0.0 ^a^	6.4 ± 2.4 ^bc^	3.1 ± 0.2 ^d^	3.4 ± 2.1 ^cd^	8.3 ± 0.6 ^ab^
Hardness (N)	0.24 ± 0.05 ^e^	1.20 ± 0.17 ^bc^	0.78 ± 0.11 ^d^	2.51 ± 0.24 ^a^	2.40 ± 0.00 ^a^	1.49 ± 0.02 ^b^	1.15 ± 0.00 ^c^	1.09 ± 0.06 ^cd^	1.00 ± 0.12 ^cd^

The values are presented as mean ± SD (*n* = 3). Data were subjected to two-way ANOVA (Table S1, factors: Flour—three levels: Control, APF and BPF degree of freedom was 2; and Thickening agent—three levels: A, G and P degree of freedom was 2; interaction “Flour × Thickening agent”; degree of freedom was 4). Different superscripts within the same row indicate significant differences of means, according to Tukey’s HSD test (*p* < 0.05).

**Table 2:** Proximate composition of jelly candies based on (**A**) agar—A, (**B**) pectin—P and (**C**) gelatin—G, with APF and BPF sweetened with erythritol, stevia, inulin and fructose (A*-APF, A*-BPF, P*-APF, P*-BPF, G*-APF and G*-BPF) in comparison with respective counterparts with sucrose (A-APF, A-BPF, P-APF¸ P-BPF, G-APF and G-BPF) and control without pomace addition (A, P and G).

(**A**)					
g/100 g	A	A-APF	A*-APF	A-BPF	A*-BPF
Fat	0.10 ± 0.02 ^b^	0.23 ± 0.04 ^a^	0.29 ± 0.05 ^a^	0.23 ± 0.04 ^a^	0.29 ± 0.03 ^a^
Proteins	0.6 ± 0.1 ^d^	1.9 ± 0.2 ^bc^	1.5 ± 0.1 ^c^	2.7 ± 0.2 ^a^	2.4 ± 0.2 ^ab^
Total CH	79.1 ± 0.2 ^a^	78.0 ± 1.1 ^ab^	77.5 ± 1.4 ^ab^	76.7 ± 1.1 ^b^	77.2 ± 1.3 ^ab^
Sugars	62.0 ± 0.6 ^b^	69.5 ± 0.7 ^a^	36.3 ± 0.6 ^c^	68.2 ± 0.9 ^a^	35.2 ± 1.0 ^c^
Glucose	10.2 ± 0.2 ^c^	16.8 ± 0.2 ^a^	3.2 ± 0.2 ^d^	14.8 ± 0.2 ^b^	2.1 ± 0.3 ^e^
Fructose	2.5 ± 0.2 ^e^	6.8 ± 0.3 ^c^	30.1 ± 0.3 ^a^	4.0 ± 0.2 ^d^	26.8 ± 0.4 ^b^
Sucrose	49.3 ± 0.3 ^a^	45.9 ± 0.5 ^b^	3.0 ± 0.3 ^d^	49.4 ± 0.5 ^a^	6.3 ± 0.3 ^c^
Total fiber	0.6 ± 0.2 ^d^	4.2 ± 0.6 ^c^	22.7 ± 0.8 ^b^	4.4 ± 0.6 ^c^	26.2 ± 0.9 ^a^
Insoluble fiber	0.41 ± 0.11 ^b^	3.1 ± 0.3 ^a^	3.7 ± 0.3 ^a^	2.9 ± 0.3 ^a^	3.4 ± 0.5 ^a^
Soluble fiber	0.10 ± 0.05 ^b^	1.1 ± 0.3 ^a^	1.0 ± 0.3 ^a^	0.9 ± 0.2 ^a^	1.0 ± 0.2 ^a^
Fructan	0.05 ± 0.01 ^c^	<0.1	18.0 ± 0.3 ^b^	0.6 ± 0.2 ^c^	21.8 ± 0.3 ^a^
Moisture	20.1 ± 0.3 ^a^	19.2 ± 0.2 ^a^	20.1 ± 0.2 ^a^	19.5 ± 0.2 ^a^	19.3 ± 0.2 ^a^
Aw	0.62 ± 0.1 ^a^	0.59 ± 0.01 ^ab^	0.57 ± 0.01 ^bc^	0.63 ± 0.01 ^a^	0.56 ± 0.00 ^c^
Ash	0.11 ± 0.04 ^b^	0.70 ± 0.12 ^a^	0.60 ± 0.1 ^a^	0.90 ± 0.2 ^a^	0.80 ± 0.2 ^a^
EV kJ/kcal	1353/319	1329/313	1149/273	1319/311	1128/269
(**B**)					
g/100 g	P	P-APF	P*-APF	P-BPF	P*-BPF
Fat	0.10 ± 0.02 ^b^	0.22 ± 0.05 ^a^	0.29 ± 0.04 ^a^	0.22 ± 0.05 ^a^	0.29 ± 0.06 ^a^
Proteins	0.6 ± 0.02 ^d^	1.2 ± 0.1 ^c^	1.8 ± 0.1 ^ab^	1.5 ± 0.2 ^bc^	2.2 ± 0.2 ^a^
Total CH	79.1 ± 0.2 ^a^	77.1 ± 1.1 ^ab^	76.6 ± 1.6 ^b^	78.0 ± 1.5 ^ab^	75.3 ± 1.3 ^b^
Sugars	65.2 ± 0.8 ^b^	69.8 ± 0.8 ^a^	35.2 ± 1.0 ^c^	69.6 ± 0.9 ^a^	35.0 ± 0.7 ^c^
Glucose	10.8 ± 0.2 ^c^	16.7 ± 0.3 ^a^	3.1 ± 0.4 ^d^	15.0 ± 0.4 ^b^	1.7 ± 0.2 ^e^
Fructose	2.9 ± 0.3 ^d^	6.6 ± 0.2 ^c^	29.5 ± 0.4 ^a^	4.1 ± 0.3 ^e^	26.6 ± 0.3 ^b^
Sucrose	51.5 ± 0.3 ^a^	46.5 ± 0.4 ^b^	2.6 ± 0.3 ^d^	50.5 ± 0.2 ^a^	6.7 ± 0.2 ^c^
Total fiber	2.2 ± 0.4 ^c^	4.3 ± 0.7 ^b^	25.7 ± 1.0 ^a^	4.9 ± 0.9 ^b^	27.6 ± 0.8 ^a^
Insoluble fiber	0.11 ± 0.05 ^c^	3.1 ± 0.5 ^b^	4.6 ± 0.4 ^a^	3.2 ± 0.4 ^b^	4.4 ± 0.4 ^a^
Soluble fiber	2.0 ± 0.3 ^a^	1.2 ± 0.3 ^ab^	1.8 ± 0.2 ^ab^	1.0 ± 0.4 ^b^	1.6 ± 0.2 ^ab^
Fructan	0.07 ± 0.03 ^d^	<0.1	19.3 ± 0.4 ^b^	0.7 ± 0.2 ^c^	21.6 ± 0.3 ^a^
Moisture	20.1 ± 0.2 ^a^	20.8 ± 0.2 ^a^	20.6 ± 0.3 ^a^	19.4 ± 0.2 ^a^	20.4 ± 0.2 ^a^
Aw	0.60 ± 0.1 ^a^	0.61 ± 0.01 ^a^	0.56 ± 0.01 ^ab^	0.61 ± 0.01 ^a^	0.55 ± 0.01 ^b^
Ash	0.10 ± 0.01 ^c^	0.71 ± 0.10 ^b^	0.70 ± 0.12 ^b^	0.90 ± 0.12 ^b^	1.8 ± 0.20 ^a^
EV kJ/kcal	1339/315	1301/307	1112/265	1316/310	1080/257
(**C**)					
g/100 g	G	G-APF	G*-APF	G-BPF	G*-BPF
Fat	<0.1	0.19 ± 0.05 ^a^	0.25 ± 0.04 ^a^	0.19 ± 0.03 ^a^	0.25 ± 0.06 ^a^
Proteins	7.8 ± 0.2 ^ab^	8.4 ± 0.1 ^a^	7.1 ± 0.2 ^b^	8.2 ± 0.1 ^a^	8.4 ± 0.3 ^a^
Total CH	71.9 ± 0.5 ^a^	71.6 ± 1.0 ^a^	71.7 ± 1.4 ^a^	72.0 ± 1.0 ^a^	70.4 ± 1.6 ^a^
Sugars	61.8 ± 0.8 ^b^	66.1 ± 0.6 ^a^	35.1 ± 0.8 ^c^	65.2 ± 0.7 ^a^	34.6 ± 1.0 ^c^
Glucose	10.1 ± 0.2 ^b^	15.0 ± 0.3 ^a^	3.1 ± 0.4 ^c^	14.7 ± 0.2 ^a^	1.9 ± 0.3 ^d^
Fructose	2.7 ± 0.2 ^d^	6.1 ± 0.2 ^c^	29.3 ± 0.4 ^a^	3.5 ± 0.2 ^d^	26.4 ± 0.4 ^b^
Sucrose	49.0 ± 0.4 ^a^	45.0 ± 0.2 ^c^	2.7 ± 0.2 ^e^	47.0 ± 0.3 ^b^	6.3 ± 0.4 ^d^
Total fiber	0.17 ± 0.1 ^d^	3.7 ± 0.5 ^c^	22.2 ± 1.0 ^b^	4.3 ± 0.6 ^c^	26.2 ± 1.0 ^a^
Insoluble fiber	0.10 ± 0.1 ^b^	2.7 ± 0.3 ^a^	3.1 ± 0.4 ^a^	2.6 ± 0.3 ^a^	3.1 ± 0.3 ^a^
Soluble fiber	<0.1	1.0 ± 0.3 ^a^	0.9 ± 0.2 ^a^	0.9 ± 0.2 ^a^	0.8 ± 0.2 ^a^
Fructan	0.07 ± 0.03 ^c^	<0.1	18.2 ± 0.4 ^b^	0.8 ± 0.1 ^c^	22.3 ± 0.5 ^a^
Moisture	20.1 ± 0.2 ^a^	19.1 ± 0.2 ^b^	20.3 ± 0.2 ^a^	18.8 ± 0.1 ^b^	20.1 ± 0.3 ^a^
Aw	0.60 ± 0.1 ^a^	0.63 ± 0.01 ^a^	0.56 ± 0.00 ^b^	0.62± 0.01 ^a^	0.60 ± 0.01 ^a^
Ash	0.21 ± 0.04 ^b^	0.7 ± 0.2 ^a^	0.7 ± 0.1 ^a^	0.8 ± 0.2 ^a^	0.8 ± 0.2 ^a^
EV kJ/kcal	1353/318	1334/314	1149/273	1332/314	1113/265

CH—CarboHydrates; EV—Energy Value; The values are presented as mean ± SD (*n* = 3). Data from all three tables, as a whole, were subjected to three-way ANOVA (Table S2, factors: Flour—tree levels: Control, APF and BPF degree of freedom was 2; Sweetener—two levels: NO-sugar* and sugar degree of freedom was 1; and Thickening agent—three levels: A, G and P degree of freedom was 2; interactions—“Flour × Sweetener” degree of freedom was 2; “Flour × Thickening agent” degree of freedom was 4; “Sweetener × Thickening agent” degree of freedom was 2; “Flour × Sweetener × Thickening agent” degree of freedom was 4). Different superscripts within the same row indicate significant differences of means, according to Tukey’s HSD test (*p* < 0.05).

**Table 3:** Ratio of EV and DF (EFR) for jelly candies prepared with pectin, agar and gelatin with substituted sucrose (marked with *) in comparison to corresponding counterparts with sucrose and controls without flour, expressed as kcal/g of dietary fiber.

	BPF *	APF *	BPF	APF	Control	14 g of DF per 1000 kcal
Pectin	9.2	10.2	61.6	70.2	142	71.4 kcal per g of food item or diet
Agar	10.3	12.0	69.5	73.6	531	
Gelatin	10.1	12.3	71.2	80.5	1744	

## Data Availability

The original contributions presented in the study are included in the article/Supplementary Material, further inquiries can be directed to the corresponding author.

## Supplementary Materials

The following supporting information can be downloaded at: https://www.mdpi.com/article/10.3390/foods14030491/s1: Table S1. Two-way ANOVA results of textural properties of jelly candies; Table S2. Three-way ANOVA results of proximate composition of jelly candies; Table S3. Three-way ANOVA results of total polyphenolics content (TPC) and AO activity of jelly candies; Table S4. Two-way repeated measures ANOVA results of OGTT (Figure 5A); Table S5. One-way ANOVA results of OGTT IAUC (Figure 5B).
