# Supplementary material for: Agro-Residues and Sucrose Alternatives in Confectionery Transformation Towards Glucose Spikes Minimization
Source: Foods. 2025 Feb 3;14(3):491. doi: 10.3390/foods14030491 (PMC11816913; doi:10.3390/foods14030491)
Supplement: Supplementary file 1 [file foods-14-00491-s001.zip › foods-3416550-supplementary.pdf]

Agro-residues and sucrose alternatives in confectionery transformation towards glucose spikes minimization

Snežana Zlatanović [1], Jovanka Laličić-Petronijević [2], Ferenc Pastor [3], Darko Micić [1], Margarita Dodevska [4], Milica Stevanović [2], Sven Karlović [5] and Stanislava Gorjanović [1,*]

[1] Institute of General and Physical Chemistry, Studentski trg 12/V, 11158 Belgrade, Serbia
[2] University of Belgrade – Faculty of Agriculture, Nemanjina 6, 11080 Belgrade, Serbia
[3] University of Belgrade – Faculty of Chemistry, Studentski trg 16, 11158 Belgrade, Serbia
[4] Institute of Public Health of Serbia »Dr Milan Jovanovic Batut«, Dr Subotica Starijeg 5, 11000 Belgrade, Serbia
[5] University of Zagreb -Faculty of Food Technology and Biotechnology, Croatia
* Correspondence: stasago@yahoo.co.uk

Table 1S. Two-way ANOVA results of textural properties of jelly candies.

	Work (mJ)	Hardness (g)	Elasticity (%)	Hardness (N)
R_c	0.9800	0.9817	0.8894	0.9817
F	110.0049	120.6396	18.0899	120.6396
Pr > F	< 0.0001	< 0.0001	< 0.0001	< 0.0001
Flour	89.4316	38.7293	1.1584	38.7293
	< 0.0001	< 0.0001	0.3363	< 0.0001
Thickening agent	282.8012	357.6127	42.2627	357.6127
	< 0.0001	< 0.0001	< 0.0001	< 0.0001
Flour × Thickening agent	33.8934	43.1082	14.4693	43.1082
	< 0.0001	< 0.0001	< 0.0001	< 0.0001

Table 2S. Three-way ANOVA results of proximate composition of jelly candies.

	Fat	Proteins	Total CH	Sugars	Glucose	Fructose	Sucrose	Total fiber	Insoluble fiber	Soluble fiber	Fructans	Moisture	Aw	Ash
R^2	0.748	0.998	0.905	0.998	0.999	1.000	1.000	0.997	0.961	0.825	0.999	0.912	0.919	0.915
F	6.405	1043.279	20.477	1179.892	1434.081	5002.772	13227.559	719.571	52.124	10.169	4205.510	22.304	25.583	23.137
Pr > F	< 0.0001	< 0.0001	< 0.0001	< 0.0001	< 0.0001	< 0.0001	< 0.0001	< 0.0001	< 0.0001	< 0.0001	< 0.0001	< 0.0001	< 0.0001	< 0.0001
Flour	16.698	154.464	3.566	122.125	1118.077	616.081	643.203	89.751	196.809	1.255	346.673	42.674	6.750	80.312
	< 0.0001	< 0.0001	0.041	< 0.0001	< 0.0001	< 0.0001	< 0.0001	< 0.0001	< 0.0001	0.301	< 0.0001	< 0.0001	0.018	< 0.0001
Sweetener	19.290	2.178	4.015	14957.220	19617.705	53384.662	147086.808	7365.246	48.596	3.922	26043.309	83.333	48.000	5.641
	0.000	0.150	0.054	< 0.0001	< 0.0001	< 0.0001	< 0.0001	< 0.0001	< 0.0001	0.058	< 0.0001	< 0.0001	< 0.0001	0.024
Thickening agent	3.021	6636.357	125.743	25.853	9.574	6.784	64.766	25.245	19.259	44.643	6.253	27.903	21.750	12.382
	0.065	< 0.0001	< 0.0001	< 0.0001	0.001	0.004	< 0.0001	< 0.0001	< 0.0001	< 0.0001	0.007	< 0.0001	< 0.0001	0.000
Flour × Sweetener	0.000	25.180	1.529	0.139	0.291	4.060	5.022	30.265	0.198	0.157		0.208	0.750	9.812
	1.000	< 0.0001	0.226	0.712	0.594	0.053	0.033	< 0.0001	0.660	0.695		0.651	0.398	0.004
Flour × Thickening agent	0.018	17.280	0.689	4.418	6.541	2.420	10.658	1.970	0.827	17.046	9.060	10.252	12.000	6.379
	0.997	< 0.0001	0.606	0.006	0.001	0.070	< 0.0001	0.125	0.518	< 0.0001	0.000	< 0.0001	0.003	0.001
Sweetener × Thickening agent	0.053	43.216	1.398	16.942	12.543	1.382	37.924	6.654	6.424	6.745	1.738	15.990	6.750	11.968
	0.948	< 0.0001	0.263	< 0.0001	0.000	0.267	< 0.0001	0.004	0.005	0.004	0.197	< 0.0001	0.018	0.000
Flour × Sweetener × Thickening agent		17.077	1.616	0.046	8.147	0.085	6.228	1.991	0.264	0.157		20.677		9.812
		< 0.0001	0.216	0.955	0.001	0.919	0.005	0.154	0.770	0.856		< 0.0001		0.001

Table 3S. Three-way ANOVA results of total phenolics (TPC) and AO activity of jelly candies.

	TPC	DPPH	FRAP
R¸	0.9853	0.9761	0.9940
F	141.7135	86.6000	350.5054
Pr > F	< 0.0001	< 0.0001	< 0.0001
Flour	674.8876	596.9590	2180.8923
	< 0.0001	< 0.0001	< 0.0001
Sweetener	133.9070	89.2452	941.8079
	< 0.0001	< 0.0001	< 0.0001
Thickening agent	415.2834	83.0771	159.0257
	< 0.0001	< 0.0001	< 0.0001
Flour × Sweetener	4.7675	9.4453	136.7264
	0.0146	0.0005	< 0.0001
Flour × Thickening agent	17.1374	0.2443	9.2679
	< 0.0001	0.9112	< 0.0001
Sweetener × Thickening agent	4.3068	0.7928	1.3546
	0.0210	0.4603	0.2709
Flour × Sweetener × Thickening agent	2.0455	0.3574	5.9286
	0.1086	0.8372	0.0009

Table 4S. Two-way repeated measures ANOVA results of OGTT (Figure 5A).

Source	DF	Sum of squares	Mean squares	F	Pr > F
Flour	2	23.1163	11.5581	8.6264	0.0067
Time	6	56.0667	9.3444	32.9518	< 0.0001
Flour × Time	12	38.6810	3.2234	11.3669	< 0.0001

Table 5S. One-way ANOVA results of OGTT IAUC (Figure 5B).

Source	DF	Sum of squares	Mean squares	F	Pr > F
Flour	2	54084.8230	27042.4115	32.2065	< 0.0001
